# Under the different sectors: the relationship between low-carbon economic development, health and GDP

**DOI:** 10.3389/fpubh.2023.1181623

**Published:** 2023-07-20

**Authors:** Shizhen Bai, Jiamin Zhou, Mu Yang, Zaoli Yang, Yongmei Cui

**Affiliations:** ^1^School of Management, Harbin University of Commerce, Harbin, China; ^2^Department of Management, Birkbeck, University of London, London, United Kingdom; ^3^College of Economics and Management, Beijing University of Technology, Beijing, China; ^4^School of Economics and Management, Beijing Jiaotong University, Beijing, China

**Keywords:** carbon neutrality, industry low-carbon economy, health, greenhouse gases, economic growth

## Abstract

Developing a modern low-carbon economy while protecting health is not only a current trend but also an urgent problem that needs to be solved. The growth of the national low-carbon economy is closely related to various sectors; however, it remains unclear how the development of low-carbon economies in these sectors impacts the national economy and the health of residents. Using panel data on carbon emissions and resident health in 28 province-level regions in China, this study employs unit root tests, co-integration tests, and regression analysis to empirically examine the relationship between carbon emissions, low-carbon economic development, health, and GDP in industry, construction, and transportation. The results show that: First, China’s carbon emissions can promote economic development. Second, low-carbon economic development can enhance resident health while improving GDP. Third, low-carbon economic development has a significant positive effect on GDP and resident health in the industrial and transportation sector, but not in the construction sector, and the level of industrial development and carbon emission sources are significant factors contributing to the inconsistency. Our findings complement existing insights into the coupling effect of carbon emissions and economic development across sectors. They can assist policymakers in tailoring low-carbon policies to specific sectors, formulating strategies to optimize energy consumption structures, improving green technology levels, and aiding enterprises in gradually reducing carbon emissions without sacrificing economic benefits, thus achieving low-carbon economic development.

## 1. Introduction

The contradiction between the environment, health, and the development of the economy has been a global concern (1, 2). Reducing carbon dioxide (CO_2_) emissions is internationally recognized as one of the effective ways to achieve sustainable development (3, 4). Since the Industrial Revolution, carbon emissions have been increasing annually with technological and economic development. Large amounts of carbon emissions result in increasing global warming problems, rising sea levels, mass extinction of species rapidly, forest fires, and the frequent occurrence of severe weather (5, 6). Due to the inevitability of economic development and the drawbacks of carbon emission, low-carbon economic development has always been an important research direction for scholars around the world (7, 8). China undoubtedly plays a significant role among carbon emitters, its energy consumption structure and efficiency improvement can greatly contribute to enhancing global environmental quality (9, 10). Currently, China’s economy is undergoing a transition toward intelligent, green, and low-carbon development (8, 11), and the urgent need for a low-carbon economy has been reflected in China’s policies (12). Previous research has demonstrated that a low-carbon economy is necessary for achieving sustainable development (13, 14). The sustainable development policies proposed by the Chinese government have achieved some positive results, such as slower growth in carbon emissions (13). However, the total carbon emissions of some sectors continue to grow, and the problem of high carbon emissions is far from being fundamentally solved. At the same time, due to differences in energy consumption types, economic development levels, and impacts on residents’ health, there are different sustainable development layouts between various sectors in China. It is worth studying how sectors control carbon emissions while promoting coordinated economic growth and improving residents’ health. Therefore, it is necessary to conduct an in-depth analysis of China’s low-carbon economic development by sectors, to understand the potential for sustainable development in different sectors.

Low-carbon economic development refers to economic development patterns that reduce the consumption of highly carbon-content energy and carbon emissions without hindering economic and social development (15). The Chinese government has made it clear that the key to improving the development of China’s low-carbon economy depends on four major industries: electricity, industry, construction, and transportation (16). Based on this, and considering our research focus, the industry, construction, and transportation sectors are identified as our research subjects. The industrial sector refers to the production activities of society that exploit natural resources and process raw materials, construction refers to civil engineering and housing construction activities, and transportation refers to the business activities of using means of transport to move goods or passengers and transfer their spatial location (17). According to the China Energy Statistical Yearbook (18), China’s total economic output and energy consumption continued to grow between 2005 and 2019. In 2005, 1.40 tons of standard coal were consumed for every 10,000 yuan of GDP growth, compared with 0.55 in 2019 (18). Although the energy consumption required to increase the total unit economy decreases, with the development of the economy, the total energy consumption increased from 250.835 million tons of standard coal to 447.597 million between 2005 and 2019, and the total carbon emissions increased by 1.81 times. Conventional wisdom holds that increased energy consumption and carbon emissions have adverse effects on health. However, previous studies have shown either mixed or inconclusive findings regarding the relationship between health, economic growth, and carbon emissions (19, 20). Therefore, this study aims to investigate the impact of low-carbon economic development, identify important gaps in carbon emissions and economic development of different sectors, and examine how the development of low-carbon economies in different sectors affects health.

We provide a conceptual discussion on the development of a low-carbon economy, which outlines clear requirements for energy consumption, carbon emissions, and economic development. Previous studies have shown a strong two-way promotion relationship between energy consumption and carbon emissions, as well as between energy consumption and economic development (21, 22). However, there seems to be no broad consensus on the relationship between carbon emissions and economic development. Some studies argue that economic development is a double-edged sword: carbon emissions increase when the economy begins to develop, but when the economy reaches a certain level, it reduces carbon emissions (23–25). Other scholars do not support this opinion, arguing that the relationship between carbon dioxide and economic development varies depending on the choice of the study regions (26, 27). The debate over the relationship between carbon dioxide and economic development continues (28). Some studies have proposed that economic development has increased the carbon emissions of countries by changing people’s consumer habits and encouraging the purchase of products with high carbon emissions. Other studies suggest that there is a different relationship between the economic development of different sectors and carbon emissions. For example, the development of the construction industry increases carbon dioxide emissions (29), while the development of tourism can have the opposite effect (30). Therefore, studying the relationship between carbon emissions of different sectors and economic development is helpful to find ways to efficiently achieve low-carbon economic development.

Compared to energy consumption, carbon emissions, and economic development, health is directly related to human beings and is an important driving force for research on low-carbon economic development. An increase in health problems can lead to disease, unemployment, and poverty (31, 32). According to the statistics of the Chinese Health Commission (33), the total per capita health expenditure in 2021 was 5348.1 yuan, and the total national health expenditure accounted for about 6.5% of GDP. Medical expenses have become a heavy burden for some families. Several studies have shown a significant relationship between health and carbon emissions (34, 35). Some suggest that carbon emissions harm health, for example, by raising the concentration of carbon dioxide (36) or the temperature (37, 38). Another partial literature argues that there is no relationship between carbon emissions and health, noting that there is no significant evidence suggesting that carbon emissions have an impact on short or long-term health (39). Additionally, after summarizing existing studies, we find that the health impacts of carbon emissions vary by sector (40, 41). For example, carbon emissions from the transportation sector mainly come from the combustion of fossil fuels, and reducing transportation can reduce the mortality rate of residents (Transport demand, harmful emissions, environment, and health co-benefits in China). The cement industry’s carbon emissions mainly come from fossil fuel combustion, the process of converting limestone and reducing the use of cement can reduce the probability of respiratory diseases. However, there is rarely research that explored the relationship between health and carbon emissions across sectors. Therefore, investigating the relationship between carbon emissions and health in different sectors can provide valuable insights into the importance of low-carbon economic development for promoting health.

Previous studies have examined the relationships between low-carbon economic development, GDP, and health. However, most scholars use a region as a whole (42–44), analyzing the relationship between the overall low-carbon economic development and other variables, ignoring the large differences between sectors. Some studies have focused on specific sectors but do not take into account the impact of the relative sectors. In this study, we analyze panel data about 28 province-level regions in China from 2005 to 2019. To examine the long-term linear validity of the model, unit root tests and co-integration tests are employed. Regression analysis is conducted to quantify the relationship between low-carbon economic development, carbon emissions, health, and GDP in the industry, construction, and transportation sectors. The results show that: First, the health impact of carbon emissions is not significant, whether in industry, construction, or transportation. Second, low-carbon economic development plays a positive and significant role in health, while the construction sector is not included. Third, carbon emissions and low-carbon economic development could increase GDP, and the improvement effect of carbon emissions is generally higher than that of low-carbon economic development. Specific to sectors, the impact of low-carbon economic development in the industry and transportation on GDP is positively significant, but the impact is not significant in the construction sector.

Through this study, we aim to make the following contributions. One of our main theoretical contributions is revealing the differences in carbon emissions and low-carbon economic development across sectors. Then, through sector-to-whole and sector-to-sector comparisons, we achieve the relationship between carbon emissions, low-carbon economic development, health and GDP, and find differences and contradictions among them. Furthermore, one of our main practical contributions is to provide references for government-industry development planning. Our findings can assist governments in identifying sectors where economic investments and the actual outcomes of energy conservation and emission reduction are disproportionate. For example, we show that despite substantial investments aimed at achieving low-carbon development, the construction industry has limited positive impacts on health and GDP. This information can inform more targeted and effective decision-making in government policies. Another practical contribution is helping industrial, construction, and transportation enterprises understand the gap between sectors that develop a low-carbon economy, and providing a clear reference for their carbon reduction goals.

## 2. Literature review

### 2.1. Low-carbon economic development

The term “low-carbon economy” first appeared in the 2003 White Paper on the Power of Government in the UK (45). Subsequently, Mantoani and Osborne (46) propose that the intensification of the global greenhouse effect would inevitably interfere with the sustainable growth of the global economy. Therefore, it is necessary to use relevant market mechanisms and government intervention to comprehensively use various energy-saving and emission-reduction technologies to achieve higher energy efficiency, lower energy consumption, and lower carbon emissions in the development of the national economy. It is from this perspective that the definition of low-carbon economic development is derived. Low-carbon economic development has become one of the important indicators to judge the efficiency of energy conservation and emission reduction in a region. Researchers have successfully used the concept of low-carbon economic development between economic development and energy consumption and carbon emission levels at the national, regional (42–44), and industrial levels (47–49).

However, measuring the development of the low-carbon economy is a complex and controversial issue. A common practice is for scholars to establish an evaluation index system to judge the development of the low-carbon economy based on the data they have and the problems to be studied. Dang et al. (50) focus on the low-carbon economic development of Guangzhou, China, and use the SEM model to establish an evaluation index system from the perspectives of energy structure and efficiency, economy, and environment. Tao et al. (51) use the LEAP model to establish an evaluation index system for low-carbon economic development based on per-capita GDP, amount of energy consumption, energy structure, and amount of CO_2_ emissions. Another approach usually appears in articles that do not focus on measuring low-carbon economic development, using existing model paradigms to judge the level of low-carbon economic development and do further research. For example, Liang et al. (52) use the Tapio decoupling model to analyze the decoupling state of Shenzhen and judge the development of the low-carbon economy by the degree of decoupling between carbon emissions and economic growth. The Tapio model calculates the ratio of changes in economic output to carbon emissions in energy-intensive sectors, and this method is also used in our study to measure low-carbon economic development. In addition, scholars have developed new machine learning algorithms to measure the development of low-carbon economies (53).

### 2.2. Influence of low-carbon economic development on health

Health is a fundamental issue in the field of social (54). According to classical economic theory, health is an important form of human capital and one of the crucial factors that promote economic development (55). In a study of health and economic development in the 20th century, Gallardo-Albarrán (56) finds that health is a significant cause of income variation during this period, particularly due to HIV, and that mortality rates increase in low- and middle-income countries. Du and You (57) propose that there is a positive and stable relationship between economic development and health; conversely, an increase in respiratory diseases can hinder economic development.

Although health contributes to economic development, it is vulnerable to influences (58, 59), such as economic and carbon dioxide. He and Qiu (40) analyze carbon emissions from different modes of consumer travel, including road, rail, waterway, and air transport. They find that reducing carbon emissions by reducing transportation volumes can also reduce deaths from related diseases due to air quality. When investigating the impact of carbon taxes on air quality, Li et al. (36) unexpectedly find that reducing carbon dioxide concentrations would also lead to fewer premature deaths. Dong et al. (37) find that carbon emissions have long-term adverse effects on human health. An increase of 1% in carbon emissions is associated with a 0.298% increase in outpatients and a 0.162% increase in inpatients, which is caused by the temperature increase resulting from increased carbon emissions. Goodman et al. (60) have another opinion, suggesting that poor health and illness are not independently related to CO^2^ emissions. Alola (39) argues that there is no significant relationship between carbon emissions and health, either in the long or short term. Sarwar et al. (19) have similar results, where their study shows that the health impact of carbon emissions differed by the calculation methodology, with carbon emissions not significant for health in the fixed effect model and difference GMM, but significant in system GMM.

Considering the relationship between health and the economy, as well as health and the environment, the impact on health cannot be ignored when studying the development of the low-carbon economy. However, few existing studies have directly analyzed the health impacts of low-carbon economic development. Instead, most explore the relationship between low-carbon economic development and health from a low-carbon perspective. For instance, Patz et al. (61) believe that low-carbon can promote future health. Haines and Dora (62) suggest that low-carbon economic development could have many benefits for health, but they acknowledge that there was no broad consensus. Therefore, when analyzing the development of the low-carbon economy, we have considered its impact on health and explored it separately from the industrial, construction, and transportation sectors.

### 2.3. Influence of low-carbon economic development on GDP

Gross Domestic Product (GDP) refers to the total value of all final products and services produced by permanent residents of a country or region within a certain period (63). It is often considered to be an important indicator for measuring the economic situation of a country or region. Due to its significance, scholars have investigated factors that affect GDP, such as human capital structure (64), the stock of education capital and fixed assets (65), and innovation factors (66). In addition to these fundamental and strategic influencing factors, low-carbon economic development also has a significant impact on GDP, which many scholars have studied in depth.

However, some scholars who study the impact of low-carbon economic development on GDP calculate the impact of carbon emissions and energy consumption on GDP to get the indirect answer. For example, Li and Li (29) analyze the carbon emissions of the construction industry and found that reducing carbon dioxide emissions and improving energy efficiency are necessary to promote GDP growth. This answers the correlation between the development of a low-carbon economy and GDP in China’s construction industry. In another study, a survey of CO_2_ emissions in China’s Yunnan Province found a significant impact between CO_2_ and GDP, the study suggests that high-carbon sectors in the region would hinder the development of low-carbon economies and affect GDP growth in the future if the capital structure was not adjusted (67). A small number of scholars directly analyze the impact of low-carbon economic development on GDP by integrating a variety of data, such as carbon emissions and energy consumption. These researches usually bring more intuitive results. For instance, Zhang et al. (68) find that the implementation of an emission trading system (ETS) has a significant positive effect on GDP when a low-carbon economy develops. Sheng et al. (69) use the Tapio model to calculate the ratio of economic growth efficiency and carbon emission reduction efficiency to estimate low-carbon economic development and the study find that it has an obvious “U”-shaped relationship with the real per capita GDP.

Despite numerous scholars investigating the relationship between low-carbon economic development and GDP, the results show significant differences. From the studies of these scholars, there are evident differences between regions and sectors, such as Samour (70) focuses on the Turkish banking industry, Li and Li (29) study on China’s construction sector, and Işık et al. (26) focuses on the top 10 cities with the highest carbon emissions in the United States. However, few scholars have explored the impact of low-carbon economic development on GDP from different sectors. Therefore, we take low-carbon economic development as a variable, starting from the perspective of multiple sectors, to explore the impact of low-carbon economic development on the GDP of China’s industrial, construction, and transportation sectors, to obtain more connections and gaps between sectors.

## 3. Materials and methods

### 3.1. Data source and descriptive statistics

The study is conducted using a panel comprising 34 provinces in China, from 2005 and 2019. Owing to the missing date, Taiwan, Macao, Tibet, Hong Kong, Chongqing, and Ningxia are removed. Different provinces in China have heterogeneity in geographical location and economic development status. Therefore, the researchers could make a thorough analysis of the problem they are trying to explain due to their variability. Most of the data comes from the China Health Statistical Yearbook (33), China Statistical Yearbook (71), and the China Energy Statistical Yearbook (18). Among the variables, the data on the development of a low-carbon economy comes from Tapio decoupling model, that is, it is determined according to the ratio of to GDP the CO_2_. This model mainly reflects the uncertain correlation between economic development, and carbon emission, and can be used to evaluate the development of a low-carbon economy (29, 72, 73). Blank data are padded by linear interpolation. Table 1 contains the definition of variables and other useful information.

**Table 1:** Variable definition.

Variables	Definition	Period
CO_2_	CO_2_ emissions (kilogram)	2005–2019
GDP	GDP of each province (hundred million)	2005–2019
LE	Low-carbon economic development	2005–2019
H	Resident health problem (incidence of tuberculosis)	2005–2019
I	Industry	
C	Construction	
T	Transportation industry	

Table 2 provides an overview of the descriptive statistics of the variables we used, covering all variables from multiple models, which can be grouped into four categories. The first category is the health status of residents, with a median of −0.155 and a difference of 7.764 between the maximum and minimum values. The second type is related to economic development, the median GDP of province-level regions is −0.278, and the range is 6.002. The third category is carbon dioxide emissions. It mainly includes CO_2_ emissions by industry. The largest median is for construction, and the largest range is also for construction. The last category is low-carbon economic development. In this category, transportation has the largest median, and the range from largest to smallest is transportation, construction, and industry.

**Table 2:** Descriptive statistics.

Variable	ZH	ZGDP	ZCO_2_	ZI_CO2_	ZC_CO2_	ZT_CO2_	ZLE	ZI_LE_	ZC_LE_	ZT_LE_
Median	−0.155	−0.278	−0.343	−0.274	−0.125	−0.149	−0.254	−0.218	−0.296	−0.130
Max	−1.517	−1.077	−1.357	−1.387	−0.497	−1.560	6.738	4.898	5.645	12.967
Min	6.247	4.925	4.023	3.459	19.065	4.109	−1.238	−1.315	−1.019	−1.077
Range	7.764	6.002	5.380	4.846	19.562	5.669	7.975	6.212	6.664	14.044

### 3.2. Model formulation

Linear regression is the main empirical tool in economics, and numerous studies have reported the significance of linear regression models (74–77). Using regression models, this study confirms the connection between low-carbon economic development, health, the growth of the economy, and the emitting of carbon, and sectors. Sarwar et al. (19) confirm the positive dependence of carbon emissions on economic development. Spiteri and Brockdorff (78) propose correspondence between health status and economic development. Given the significant long-term impact of the economy on health, we exclude the influence of GDP according to the Frisch-Waugh-Lovell theorem when calculating the relationship between carbon emissions, low-carbon economic development and health (79, 80). Building on previous research, we try to introduce industry, construction, and transportation, and examine the connections and differences between variables in the different sectors. For example, how do carbon emissions affect health and economic development? Is a low-carbon economy essential for health and the GDP of different sectors? To answer these questions, we highlight two dependent variables with four models.


(1) $$H_{it}=\beta_{0}+\beta_{1}CO_{2}+\mu_{it}$$

(2) $$H_{it}=\beta_{0}+\beta_{1}ZI_{CO_{2}}+\beta_{2}ZC_{CO_{2}}+\beta_{3}ZT_{CO_{2}}+\mu_{it}$$

(3) $$H_{it}=\beta_{0}+\beta_{1}LE+\mu_{it}$$

(4) $$H_{it}=\beta_{0}+\beta_{1}ZI_{LE}+\beta_{2}ZC_{LE}+\beta_{3}ZT_{LE}+\mu_{it}$$

(5) $$GDP_{it}=\beta_{0}+\beta_{1}CO_{2}+\mu_{it}$$

(6) $$GDP_{it}=\beta_{0}+\beta_{1}ZI_{CO_{2}}+\beta_{2}ZC_{CO_{2}}+\beta_{3}ZT_{CO_{2}}+\mu_{it}$$

(7) $$GDP_{it}=\beta_{0}+\beta_{1}LE+\mu_{it}$$

(8) $$GDP_{it}=\beta_{0}+\beta_{1}ZI_{LE}+\beta_{2}ZC_{LE}+\beta_{3}ZT_{LE}+\mu_{it}$$

where $\beta_{1}$ , $\beta_{2}$ , and $\beta_{3}$ are the coefficients of variables, while $\mu_{it}$ is the error term and the distribution of it conforms to the normal distribution with a mean of zero. Also, i (i = 1, 2, 3..., N) represents the inquired provinces, while t (t = 1, 2, 3..., T) means the change in the time frame. At last, $\beta_{0}$ represents the constant. To help minimize heteroscedasticity and undulation in data, all of the variables in equations were standardized by zero-mean normalization.

## 4. Results

### 4.1. Unit root tests of augmented dickey-fuller test

The data used by the model is time series, to prevent the occurrence of spurious regression, we inspect the panel unit root of all variables using the ADF test, assuming a unit root in the null hypothesis. This is done before conducting the panel regression models. It is shown in Table 3, the results of the ADF test statistic for almost all sequence variables are less than the cut-off value of 5% significance level, the rejection of the null hypothesis of the ADF test indicates that the time series data is stationary. However, there is an exception, the variable Health is non-stationary, so we further perform second-order differential on the data, and after that, all variables become stationary.

**Table 3:** The values of the ADF test.

Variable		Differential order	*t*	*P*	AIC	Critical value			Results
						1%	5%	10%	
H	ZH	0	−1.31	0.625	498.024	−3.447	−2.869	−2.571	Non-stationary
		1	−5.905	0.000***	497.69	−3.447	−2.869	−2.571	Stationary
GDP	ZGDP	0	−3.911	0.002***	530.563	−3.447	−2.869	−2.571	Stationary
		1	−5.379	0.000***	543.866	−3.447	−2.869	−2.571	Stationary
CO_2_	ZCO_2_	0	−3.955	0.002***	384.852	−3.447	−2.869	−2.571	Stationary
		1	−5.217	0.000***	398.962	−3.447	−2.869	−2.571	Stationary
	ZI_CO2_	0	−3.951	0.002***	357.786	−3.446	−2.868	−2.57	Stationary
		1	−20.776	0.000***	373.265	−3.446	−2.868	−2.57	Stationary
	ZC_CO2_	0	−12.31	0.000***	1148.711	−3.446	−2.868	−2.57	Stationary
		1	−9.156	0.000***	1191.975	−3.447	−2.869	−2.571	Stationary
	ZT_CO2_	0	−4.107	0.001***	557.938	−3.447	−2.869	−2.571	Stationary
		1	−5.969	0.000***	572.192	−3.447	−2.869	−2.571	Stationary
LE	ZLE	0	−3.612	0.006***	328.369	−3.447	−2.869	−2.571	Stationary
		1	−5.815	0.000***	338.418	−3.447	−2.869	−2.571	Stationary
	ZI_LE_	0	−3.825	0.003***	393.318	−3.447	−2.869	−2.571	Stationary
		1	−5.659	0.000***	404.537	−3.447	−2.869	−2.571	Stationary
	ZC_LE_	0	−4.864	0.000***	527.88	−3.446	−2.869	−2.571	Stationary
		1	−10.493	0.000***	546.66	−3.446	−2.869	−2.571	Stationary
	ZT_LE_	0	−3.18	0.021**	1005.941	−3.447	−2.869	−2.571	Stationary
		1	−5.363	0.000***	998.181	−3.447	−2.869	−2.571	Stationary

*p < 0.05; **p < 0.01; ***p < 0.01.

### 4.2. Co-integration test

Cointegration tests help us determine whether the data exhibits a long-term stable relationship between a set of linear data. Consequently, we further examine the cointegration relationship of the linear regression model and perform a stationary analysis of the model residual sequence. As shown in Table 4, the residual sequence test results for models 1–6 are all less than the 5% cut-off value, so the residual sequences of these models are stable. Thus, the independent and dependent variables in this study are found to be co-integrated, indicating a long-term correlation between them.

**Table 4:** The residual sequence test results for models 1–6.

Variable	Differential order	*t*	*P*	AIC	Critical value			Results
					1%	5%	10%	
The residuals of model 1	0	−2.573	0.099*	430.985	−3.446	−2.868	−2.57	Stationary
	1	−6.608	0.000***	435.244	−3.447	−2.869	−2.571	Stationary
The residuals of model 2	0	−2.588	0.095*	437.674	−3.446	−2.868	−2.57	Stationary
	1	−5.861	0.000***	441.014	−3.447	−2.869	−2.571	Stationary
The residuals of model 3	0	−2.688	0.076*	425.04	−3.446	−2.868	−2.57	Stationary
	1	−16.65	0.000***	429.483	−3.446	−2.869	−2.57	Stationary
The residuals of model 4	0	−2.534	0.107	463.697	−3.446	−2.869	−2.57	Non-stationary
	1	−17.407	0.000***	467.751	−3.446	−2.869	−2.57	Stationary
The residuals of model 5	0	−2.698	0.074*	476.741	−3.447	−2.869	−2.571	Stationary
	1	−5.778	0.000***	482.033	−3.447	−2.869	−2.571	Stationary
The residuals of model 6	0	−3.888	0.002***	640.956	−3.447	−2.869	−2.571	Stationary
	1	−6.095	0.000***	653.993	−3.447	−2.869	−2.571	Stationary
The residuals of model 7	0	−4.44	0.000***	495.528	−3.447	−2.869	−2.571	Stationary
	1	−5.712	0.000***	513.914	−3.447	−2.869	−2.571	Stationary
The residuals of model 8	0	−5.654	0.000***	605.482	−3.446	−2.868	−2.57	Stationary
	1	−6.645	0.000***	627.913	−3.447	−2.869	−2.571	Stationary

*p < 0.05; **p < 0.01; ***p < 0.01.

### 4.3. Regression analysis

Table 5 reports the relationship between health and carbon emissions or low-carbon economic development with the low-carbon economic development of industry, construction, and transportation, as shown in Equations 1–4. Table 6 reports the correlation in GDP, carbon emissions and low-carbon economic development of the three sectors, as shown in Equations 5–8.

**Table 5:** Model results with resident health problem as the dependent.

Dependent variable	Independent variable	Model 1	Model 2	Model 3	Model 4
H	CO_2_	−0.082			
	I_CO2_		0.070		
	C_CO2_		−0.042		
	T_CO2_		−0.061		
	LE			−0.168***	
	I_LE_				−0.194***
	C_LE_				−0.031
	T_LE_				−0.104*

*p < 0.05; **p < 0.01; ***p < 0.01.

**Table 6:** Model results with GDP as the dependent variable.

Dependent variable	Independent variable	Model 5	Model 6	Model 7	Model 8
GDP	CO_2_	0.605***			
	I_CO2_		0.323***		
	C_CO2_		0.050*		
	T_CO2_		0.619***		
	LE			0.533***	
	I_LE_				0.582***
	C_LE_				−0.052
	T_LE_				0.183***

*p < 0.05; **p < 0.01; ***p < 0.01.

For both Model 1 and Model 2, health is the dependent variable. The independent variable of Model 1 is overall carbon emissions, and our study finds that carbon emissions have no significant effect on overall health. The independent variables of Model 2 are carbon emissions from industry, construction, and transportation. Our results suggest that the health impact of carbon emissions in either sector is not significant, meaning that increased carbon emissions do not lead to more health problems directly.

Models 3 and 4 report the health impacts of low-carbon economic development, especially in the industry, construction and transportation sectors. As revealed by our research, the implementation of low-carbon measures in industry and transportation has a notable positive effect on health, while the construction sector is not. These two model results show that a strong low-carbon economy can help reduce health problem occurrence, especially in industry and transportation. Combining the results of models 3 and 4, we note the unusual nature of the industry. We found that the low-carbon progress of industry has a greater effect on reducing health problems than other sectors. This shows that industry has a greater impact on health than construction and transportation sectors. Industrial enterprises must pay more attention to the growth of the low-carbon economy.

For model 5, the findings from the linear regression analysis revealed a statistically significant positive correlation between emitting of carbon and regional GDP. Model 6 is an extension of Model 3 to further categorize carbon emissions into industrial, construction and transportation. It demonstrates that the carbon emissions of industry and transportation have a substantial beneficial impact on the regional GDP, while the emitting of carbon in the construction industry is not significant, which may be related to the unique features of the construction industry itself. The different impacts we observe on economic development from different sectors synthesize the results of some studies (43, 44, 81).

Models 7 and 8 illustrate a significant effect of low-carbon economic development on GDP. Derived from the outcomes of Model 7, low-carbon economic development has a considerable positive effect on regional GDP. Model 8 shows the impact of low-carbon economic development in industry, construction, and transport on GDP. We find that industry and transportation have a positive and significant influence, meantime, the impact of low-carbon economic progress in the construction industry is not significant, that is, from the overall point of view, the growth of the low-carbon economy will affect the collectivity economic progress, and by industry, the growth of the low-carbon economy in the construction industry has nothing to do with the overall economic development, and the industrial and transportation industry is consistent with the overall situation.

Combining the results of Models 6 and 8, we believe that both carbon emissions and low-carbon economic development can have a significant beneficial influence on regional economic development. For industry, low-carbon economic development owns a greater influence on the regional development of the economy than emitting carbon. Neither carbon emissions nor low-carbon economic development in the construction industry has a substantial beneficial effect on regional economic development. For the transportation industry, carbon emissions have a greater influence on regional economic development than low-carbon economic development. In summary, the reduction of emitting of carbon in industries will not bring about a reduction to every layer of the growth of the economy, although the effect of carbon emission reduction in other sectors on the economy is not as significant as that of industry, it will not have a negative impact.

## 5. Discussion and conclusion

### 5.1. Discussion

Low-carbon economic development is a crucial and effective approach for achieving sustainable economic growth and improving people’s health. Our results indicate that carbon emissions have no direct impact on health but have a positive impact on GDP. However, low-carbon economic development has a positive effect on both health and GDP. Our findings align with some existing literature, showing that carbon emissions do not have a significant impact on health (38), but can be improving economic development (39) and that low-carbon economic development can simultaneously enhance human health and economic prosperity (61). Our research shows that the development of a low-carbon economy can bring multiple benefits, not only improving economic development but also promoting the residents’ health.

The results of this study show that carbon emissions have no significant impact on health. Our conclusions are inconsistent with some previous studies suggesting that carbon emissions have significant negative effects on health (34, 82, 83). On the one hand, carbon emissions bring about a greenhouse effect, harming health by raising temperatures (38, 39). On the other hand, other harmful pollutants that accompany carbon emissions during the combustion of fossil fuels could harm health (35, 84). Goodman et al. (60) argues that health and carbon emissions are not independently correlated unless the environment is used as an intermediary. Our research directly links carbon emissions to residents’ health and does not involve environmental impacts. Goodman’s results have provided a good explanation for the differences between our results and some literature. Our findings also demonstrate that there is no direct relationship between carbon emissions and health in the industrial, construction, and transportation sectors. Guo et al. (41) investigate the health effects of the Chinese industry and find that particulate matter (PM) emissions accompanying carbon emissions are the main cause of health damage. Zhang et al. (85) also obtain similar results in their investigation of the relationship between energy, emissions, and health in the construction sector, finding that PM and air pollution are the main causes of health problems. In the transportation sector, the incomplete combustion of fossil fuels releases large amounts of carbon dioxide and brings various pollutants (86, 87), and the use of fossil fuels poses a significant health risk (88, 89). He and Qiu (40) propose in their investigation of the relationship between carbon emissions and health in the transportation sector in China that reducing the use of fossil fuels can reduce the pollution that accompanies carbon emissions and thereby reduce health problems. These studies support Goodman’s (60) view that health and CO_2_ emissions are not independently correlated, and further support our finding that there is no direct significant relationship between carbon emissions and health.

Our research shows that the development of a low-carbon economy has a positive and significant impact on health, this finding is supported by previous research (36). Broken down to specific sectors, the health impacts of low-carbon economic development are markedly different, it still shows positive and significant impacts in industry and transportation, but not significant in the construction sector. The reasons for this outcome may be linked to GDP and carbon emissions, which are commonly used to measure the progress of low-carbon economic development (29, 72, 73). When neither GDP nor carbon emissions have an impact on health, it is unlikely that the promotion of low-carbon economic development will significantly influence health. This finding is consistent with our results in the construction sector, which show that neither carbon emissions nor GDP have a significant effect on health. Further investigation into the underlying mechanism of this finding leads us to believe that the type and quantity of energy consumption remain crucial factors, and the coupling effect between GDP and carbon emissions should not be disregarded. When GDP and carbon emissions are weakly coupled, meaning that energy conservation and emission reduction efforts cannot achieve low-carbon economic development (73), there naturally is no significant impact on health.

It has been found that carbon emissions have a significant positive impact on GDP, which is supported by previous studies (21, 22). However, some studies have shown that carbon emissions do not necessarily promote GDP when the economy has reached a certain level of development (23–25). We believe that the reasons for the differences in research results may be related to the selected regions and countries, as they have significant differences in economic development level and industrial structure (26, 27). When comparing the impact of carbon emissions on GDP across different sectors, our results show that the impact of industry and transportation is significantly greater than that of construction. Previous studies have shown an increasing dependence of the transportation sector development on CO_2_ emissions, and carbon emissions play a significant role in promoting economic development (90), while Li and Li (29) propose that carbon emissions have less effect on economic development in the construction sector. We think that the reasons for the differences between sectors may be related to population, energy structure, and energy efficiency (29, 90). In addition, the level of industry development may also be the main reason for the difference, and scholars usually use economic and social indicators for comprehensive measurement (91–93). Some studies have shown economic development and carbon emissions are linked through the Environmental Kuznets Curve (EKC)^1^ mechanism (94–96). Currently, China’s carbon emissions are increasing with economic growth, within the climbing stage of the mechanism’s inverted U-shaped curve. Our findings on the impact of carbon emissions on economic development support this view. The situation in the industrial and transportation sectors should be closer to the initial stage of EKC, while the construction industry should be closer to the inflection point.

The results demonstrate that low-carbon economic development has a significant, positive impact on GDP, as documented in previous literature (67, 68). Promoting economic growth is an important prerequisite for developing a low-carbon economy, as low-carbon economic development can significantly increase a country or region’s gross domestic product. Du et al. (97) discover a positive correlation between the level of economic development in most provinces in China and carbon emissions. However, some cities are experiencing decoupling. Decoupling implies that economic development no longer leads to increased carbon emissions, and efforts to reduce carbon emissions do not hinder economic development, such as Guangdong and Jiangsu (73, 98), which have achieved higher levels of economic development and lower carbon emissions through improved energy efficiency and the development of information technology (73, 99). These findings are consistent with our findings. In our survey across various sectors, we observed that the development of a low-carbon economy in the industrial and transportation sectors has a significant positive impact on GDP. However, we did not find a significant impact of low-carbon development in the construction sector on GDP. In the industrial sector, there is a weaker decoupling relationship between carbon emissions and GDP (100, 101), indicating that carbon emissions are growing at a slightly slower rate than GDP. Similar results were found in the transportation sector (102, 103). However, in the construction sector, Zhang et al. (104) find diverse decoupling relationships between carbon emissions and economic development, with most provinces exhibiting either strong or weak decoupling and evident spatial heterogeneity. Consequently, low-carbon economic development in the construction industry does not significantly impact GDP. Further analysis suggests that the level of industrial economic development may be a contributing factor. Additionally, the source of carbon emissions could also explain the variations between industries. While carbon emissions in the industrial and transportation sectors mainly result from the use of fossil fuels, the construction industry’s carbon emissions come not only from fossil fuel consumption but also from activities involving concrete and dump trucks (105, 106).

### 5.2. Conclusion

This study uses regression models to discover the relationship between health, GDP, and low-carbon economic development. To this end, we used data from 28 province-level regions in China between 2005 and 2019 to further categorize data on low-carbon economic development, carbon emissions, and regional GDP according to industry, construction, and transportation, delved into it to obtain cross-industry findings. The ADF root test and co-integration test were used to verify the model’s validity and the long-term stable relationship between the data. The model results report the positive significant impact of low-carbon economic development on health and GDP. However, when it comes to specific sectors, these impacts may not be significant. We note that the influence of low-carbon economic development on health and GDP varies significantly between different sectors.

This study has important theoretical implications. First, we survey the development of low-carbon economies across the whole and by sectors, and most previous studies have ignored comparisons between sectors and rarely covered differences between sectors and the whole. Comparing sectors can provide valuable insights into data analysis and help us understand relative-level relationships between phenomena. Furthermore, comparing sectors to the overall economy allows us to examine the relationship between the whole and its parts, thereby uncovering differences and contradictions. These sector comparisons enable us to identify bottlenecks and shortcomings in low-carbon economic development and devise targeted solutions to address them. Secondly, by discussing the impact of carbon emissions and the development of a low-carbon economy on health, it is clear that the development of a low-carbon economy can contribute to the improvement of health issues. By reducing reliance on fossil energy and enhancing energy efficiency, low-carbon economic development can effectively reduce carbon emissions, improve the national economy and promote the health of residents. Conducting horizontal comparisons across sectors allows us to identify sectors that have a significant impact on human health, enabling us to address and mitigate potential health hazards. Taking sectors that can promote human health, such as industry and transportation, as a reference, guiding improve residents’ health, which has not been covered in most previous studies focusing on region (47) or single (48) sectors. Previous studies on the health or economic impacts of low-carbon economic development often did not indicate their direct impact but used carbon emissions as an intermediate means to reach conclusions (67). This study uses regression models to reveal the direct impact of low-carbon economic development on health or the economy, which helps to reach clearer conclusions. In addition, the study expands the understanding of the health or economic impacts of low-carbon economic development in different industries, complementing previous research results based on regions (26, 29) or single sectors (70).

This study has important practical implications and serves as a reference for governments and enterprises. Our research findings highlight the critical role of carbon emissions in economic development. We have demonstrated that low-carbon economic development is a key pathway toward achieving sustainable development. The Chinese government should prioritize low-carbon and sustainable economic development, continue implementing low-carbon measures, and accelerate the transformation and upgrading of the industrial structure. The results of industrial data comparison point out that in the industrial and transportation sectors, carbon emissions and low-carbon economic development both play a positive role in GDP. Therefore, in the industrial sector, the government should gradually increase investment in science and technology, accelerate industrial upgrading, reduce energy consumption, improve energy efficiency, and develop zero-carbon factories. In the transportation sector, the government should optimize the transportation structure, encourage the use of new energy vehicles, strengthen people’s environmental education, and improve awareness of low-carbon and green travel. For the construction sector, since the development of a low-carbon economy does not have a significant role in GDP at present, the Chinese government should make appropriate adjustments based on good practices in the industrial and transportation sectors and propose targeted low-carbon measures for construction consumables and large loading vehicles. Our findings also show that low-carbon economic development in industry and transportation can have a positive effect on health. We suggest that the Chinese government should focus on sectors where industry and transportation play a significant role in improving health, advocate for the reduction of fossil fuel usage, increase investment in new energy sectors, proposes incentive policies for energy conservation and emission reduction, encourages residents to travel in a green and healthy manner, and ultimately aim to improve resident health.

Furthermore, this study will help industrial, construction, and transportation enterprises understand the differences between low-carbon economic development sectors, enhance their awareness of low-carbon development, and provide a clear reference for their carbon emission reduction goals. Enterprises can establish energy monitoring and control centers to improve the sustainable growth of energy conservation and emission reduction with automation and informatization. They can also do a good job in the transformation and application of new energy technologies, and achieve low-carbon economic development through effective supervision and the use of infrastructure.

There are several limitations to this study. Firstly, the data used in this study are limited to China. Although China is a major carbon emitter, the results cannot be generalized to other countries and regions due to the differences in their levels of economic development. Future research on low-carbon economic development should survey more representative countries or even the world, and determine the differences in the impact of low-carbon economic development on health and GDP in countries or regions with different levels of economic development. Secondly, when investigating the impact of low-carbon economic development on GDP in different sectors, this study only focuses on industry, construction, and transportation, as they are important sectors that affect the country’s economy and carbon emissions (107). Future research could investigate the development of low-carbon economies in more specific sectors, such as food manufacturing and textiles, which are also important concerns for national sustainable development. Additionally, further investigation is needed to explore the specific impact of carbon emissions and low-carbon economic development on health across different sectors. This is crucial as health is an essential requirement for promoting comprehensive human development and serves as a fundamental condition for economic and social progress (108). Future research should prioritize the investigation of this aspect, expanding the scope to include more regions and countries, as well as examining specific sectors in more detail, such as heavy industry and light industry.

## Data availability statement

The original contributions presented in the study are included in the article/Supplementary material, further inquiries can be directed to the corresponding author.

## Author contributions

SB: conceptualization, methodology, software, investigation, writing—original draft, and funding acquisition. JZ: investigation, data curation, and writing—original draft. MY: conceptualization, resources, supervision, writing—review and editing. ZY: resources and supervision. YC: software and validation. All authors contributed to the article and approved the submitted version.

## Funding

This research was supported by three funding: The Ministry of Education funded major projects in the later stage of philosophy and social sciences (Bai Shizhen; 22JHQ009); The central government supports the reform and development of high-level talent projects in local colleges and universities (Bai Shizhen; 2020GSP13); Natural Science Foundation of Heilongjiang Province (Bai Shizhen; LH2021G014).

## Conflict of interest

The authors declare that the research was conducted in the absence of any commercial or financial relationships that could be construed as a potential conflict of interest.

## Publisher’s note

All claims expressed in this article are solely those of the authors and do not necessarily represent those of their affiliated organizations, or those of the publisher, the editors and the reviewers. Any product that may be evaluated in this article, or claim that may be made by its manufacturer, is not guaranteed or endorsed by the publisher.

## Footnotes

^1^Environmental Kuznets Curve (EKC) proposed by Panayotou in 1993, which is an inverted U-shape curve reflect the relationship between environmental quality and per capita income.
